# Influence of nutritional status on rehabilitation efficacy of patients after stroke—a scoping review

**DOI:** 10.3389/fneur.2025.1502772

**Published:** 2025-01-29

**Authors:** Huan Chen, Caihong Fu, Weihao Fang, Zhenyao Wang, Dawei Zhang, Hu Zhang

**Affiliations:** Department of Rehabilitation, Shunyi Hospital, Beijing Hospital of Traditional Chinese Medicine, Beijing, China

**Keywords:** proteins, vitamin D, essential amino acids, antioxidants, nutritional supplement, rehabilitation, stroke

## Abstract

Stroke patients are susceptible to malnutrition as a result of dysphagia, neurological impairments, and altered levels of consciousness. The nutritional status of individuals post-stroke is a critical determinant influencing the efficacy of rehabilitation outcomes. Therefore, there is great interest in the possible role of nutrients in promoting recovery after stroke. This article examines the enhancement of rehabilitation outcomes through the improvement of nutritional status. A comprehensive literature search was conducted using the PubMed, Cochrane, Web of Science, and Embase databases. Articles relevant to this topic, published from the inception of each database until November 2024, were identified. The selection was restricted to randomized controlled clinical trials, irrespective of language or publication date. The search specifically targeted studies involving stroke patients, encompassing both hemorrhagic and ischemic types, and interventions that combined nutritional supplementation with rehabilitation therapy. Studies with a focus on stroke prevention were excluded. Full-text articles that met the inclusion criteria were retrieved from the aforementioned sources. In instances where both a full report and a conference abstract were available for the same study, only the full report was considered. A total of 751 studies were considered for inclusion in this scoping review. Following a rigorous screening process, 13 studies were selected for detailed analysis. All selected studies were randomized controlled clinical trials. The findings indicate that supplementation with nutrients such as proteins, vitamins, essential amino acids, and antioxidants can enhance activities of daily living, improve balance function, and reduce neurological deficits in post-stroke patients. This review aims to synthesize current evidence regarding the effects of various nutrients and dietary regimens on limb rehabilitation in post-stroke patients, with the goal of providing new insights to facilitate the accelerated recovery of this population.

## 1 Introduction

Globally, one in four people will have a stroke in their lifetime, making strokes the second most common cause of death and the third most common cause of disability (1, 2). Since 2015, stroke has become the leading cause of death and disability in China. As a major chronic non-communicable disease, it poses a serious threat to national health. Over the past decade, stroke prevalence, recurrence, and mortality in China have steadily increased (3).

According to Global Burden of Disease (GBD) data, the prevalence of ischemic stroke in China increased from 1,100 per 100,000 individuals in 2010 to 1,256 per 100,000 individuals in 2019. The 12-month stroke recurrence rate among stroke survivors was reported to be 5.7%. The “China Health Statistics Yearbook 2020” indicates that the crude mortality rate of stroke in China in 2019 ranged from 129.41 to 158.63 per 100,000 individuals (3). The average age of stroke patients in China is approximately 65 years, which is notably lower than the average age of around 75 years in developed countries (4). Additionally, malnutrition is prevalent among elderly Chinese stroke patients and is associated with increased mortality (5). Malnutrition negatively impacts the rehabilitation outcomes of patients recovering from stroke and hinders the enhancement of their daily living abilities. Addressing malnutrition is essential for stroke recovery, as improving nutritional status may contribute to the rehabilitation process. Nevertheless, a direct causal relationship between nutritional status and rehabilitation efficacy has yet to be definitively established. To investigate whether enhancing the nutritional status of post-stroke patients could potentially improve rehabilitation outcomes, we conducted this scoping review.

### 1.1 Stroke patients at risk of malnutrition

Studies have found that both acute stroke patients and stroke survivors have certain nutritional risks and deficiencies. Based on the third national stroke registry data, 1.95 to 5.89% of acute stroke patients in China had moderate to severe malnutrition risk. The risk of malnutrition in acute stroke patients is associated with an increased risk of long-term death and severe disability (6). A randomized controlled trial (RCT) involving 323 participants demonstrated that the incidence of anemia among stroke patients during the recovery phase was 42.4%. Additionally, the incidence rates of total protein, albumin, and prealbumin levels falling below normal were 8, 17, and 31.9%, respectively (7). These findings indicate that stroke patients experience significant nutritional deficiencies. Furthermore, a meta-analysis encompassing 915 subjects revealed that malnutrition is particularly prevalent in patients with ischemic stroke and is independently correlated with an elevated risk of stroke-related pneumonia (8).

### 1.2 Stroke patients with malnutrition may have a poor prognosis

Malnutrition is an independent risk factor for poor prognosis in stroke patients (9). There are many problems associated with malnutrition, including imbalances in energy, protein, vitamins and minerals, loss of self-care ability, prolonged hospitalization, poor functional prognosis, and increased mortality (10, 11). Gomes et al. (12) determined that the risk of malnutrition, as assessed by the Malnutrition Universal Screening Tool (MUST), serves as an independent predictor of mortality, service level, and hospitalization costs within 6 months post-stroke. The most apparent manifestation of malnutrition is weight loss. Specifically, a weight reduction exceeding 3 kg following a stroke significantly elevates the risk of mortality in both the short and long term. Early intervention in such cases can substantially enhance clinical outcomes for patients (13). Nishioka et al. (14) conducted a cross-sectional survey involving 178 stroke recovery patients aged 65 and above, revealing that the enhancement of nutritional status in elderly stroke patients experiencing malnutrition during the recovery phase is associated with significant improvements in daily living activities. Similarly, in patients experiencing acute stroke, malnutrition has been found to be negatively correlated with activities of daily living (ADL) (15).

The extent of neurological deficits following a stroke is positively correlated with the degree of subsequent nutritional deterioration. This decline in nutritional status impedes neurological recovery, elevates the incidence of complications, increases hospitalization costs, and extends the duration of hospital stays (16). A RCT study (*n* = 277) found that the Geriatric Nutrition Risk Index (GNRI) score at admission was closely related to the neurological function 3 months after stroke. The higher the GNRI nutritional risk level, the worse the neurological prognosis in the recovery period (17).

### 1.3 Stroke patients’ risk factors for malnutrition

Numerous risk factors contribute to malnutrition in stroke patients, including swallowing disorders, neurological deficits, impaired consciousness, advanced age, female sex, pre-existing malnutrition, suboptimal family conditions or inadequate care, the presence of malignant tumors, delayed initiation of rehabilitation, and a history of severe alcoholism (18–20). Additionally, factors such as polypharmacy, feeding difficulties, chronic diseases, functional impairments, and elevated National Institutes of Health Stroke Scale (NIHSS) scores at admission are correlated with an increased risk of malnutrition in this patient population. Dysphagia is identified as the primary risk factor for malnutrition in patients who have experienced a stroke (12).

## 2 Methods

The Preferred Reporting Items for Systematic Reviews and Meta-Analyses (PRISMA) Extension for Scoping Reviews statement was followed to report in this study.

### 2.1 Search strategy

An exhaustive search of the literature was conducted by a medical librarian (FWH) in the Cochrane Library, Ovid Embase, PubMed and Web of Science Core Collection databases to find relevant articles published from earliest database record to 25 November 2024. Databases were searched using a combination of controlled and free text terms for Strokes/Cerebrovascular Accidents, Nutrients, rehabilitation. Randomized controlled clinical trials were exclusively selected for inclusion, regardless of the language or date of publication.

### 2.2 Study selection

**Figure 1:**
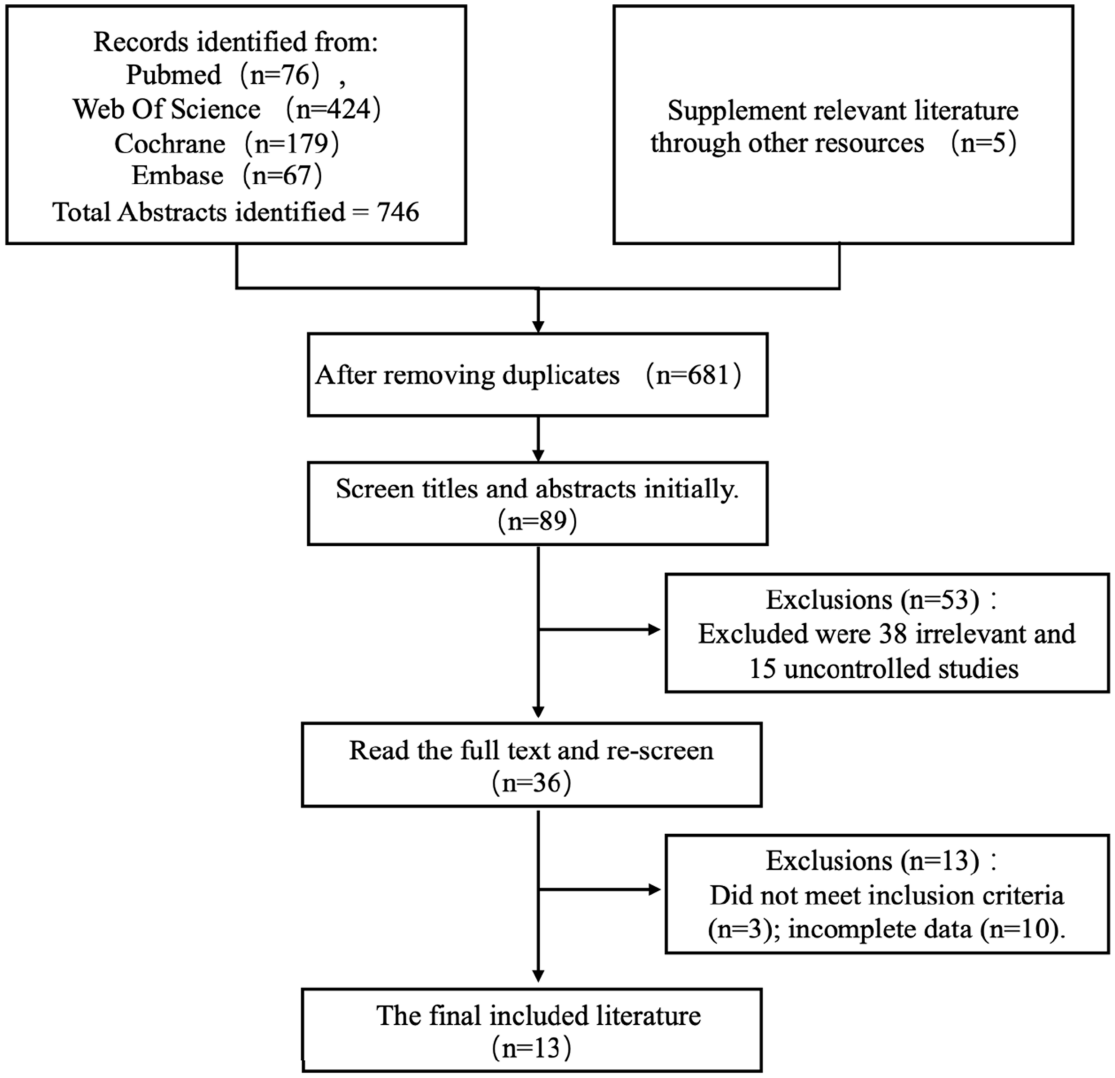
PRISMA flow chart diagram describing the study selection process.

Citations from all databases were imported into the Endnote 20 library (Clarivate Analytics, Philadelphia, PA, United States). Two reviewers (CH and FCH) meticulously evaluated the collected titles and structured abstracts based on predefined criteria. The search was confined to studies involving patients who had experienced a stroke, encompassing both hemorrhagic and ischemic types, and interventions that integrated nutritional supplementation with rehabilitation therapy. Studies focused on stroke prevention were excluded. Complete articles that satisfied the selection criteria were obtained from the aforementioned sources. No exclusion criteria were applied regarding language or year of publication. In instances where both a full report and a conference abstract were available for the same study, only the full report was included. The suitability of articles for the final review was determined by two reviewers, with any disagreements resolved through consultation with a third reviewer (ZH). The selection process was guided by a PRISMA flow diagram (Figure 1).

Our search initially identified 751 titles. Following the removal of duplicates, 681 studies underwent detailed examination. From these, 36 reports, comprising both articles and abstracts, were selected for further consideration, and ultimately, 13 randomized controlled clinical trials were included in the final review. Three reviewers (CH, FCH, FWH) extracted and summarized the data into a table (Table 1).

**Table 1:** Summary of studies reporting the effects of nutritional supplements on rehabilitation outcomes in stroke patients.

Authors, Year	Design	Nutritional supplement	Number of participants	Age (years)		Stage of stroke	Rehabilitation program	Duration of intervention (week)
				Intervention	Control			
Aquilani R et al., 2008 (21)	RCT	Protein	41	71 ± 6.9	68 ± 9.1	Acute phase	Standard rehabilitation therapy consisting of proprioceptive neuromuscular facilitation (90 min/day over 5 days weekly).	3
Rabadi MH et al., 2008 (22)	RCT	Protein	116	75.0 ± 10.9	73.6 ± 13.0	Subacute phase	Rehabilitation therapies (physical, occupational, speech)	2
Cheng YH et al., 2020 (23)	RCT	Protein	18	58.8 ± 7.1	56.8 ± 10.1	Recovery phase	Alongside regular PT and OT, three weekly 40 min cycling ergometry training.	8
Sakai K et al., 2019 (24)	Meta-analysis of 8 RCTs	Protein	5,484	–	–	Subacute and recovery phase	Routine rehabilitation protocols (Not specifically described)	
Aylin Sari, 2018 (25)	RCT	Vitamin D	67	69.8 ± 10.1	66.9 ± 10.1	Recovery phase	Rehabilitation therapies (physical, occupational, speech)	12
Shuba Narasimhan, 2017 (26)	RCT	Vitamin D	60	62	65	Not described	Routine rehabilitation protocols (Not specifically described)	12
Momosaki R et al., 2019 (27)	RCT	Vitamin D	97	67.6 ± 11.7	65.5 ± 11.7	Recovery phase	Routine rehabilitation protocols (Not specifically described)	8
Torrisi M et al., 2021 (28)	RCT	Vitamin D	19	59.20 ± 11.4	62.1 ± 10.8	Recovery phase	Motor modules included four daily rehabilitative sessions, 6 days a week, lasting about an hour each. Cognitive rehabilitation included daily sessions based on exercises focused on enhancing attention and memory abilities	12
Honaga K et al., 2022 (29)	RCT	Vitamin D	50	64.2 ± 8.9	61.3 ± 11.5	Recovery phase	Routine rehabilitation protocols (Not specifically described)	16
Yoshimura Y et al., 2019 (31)	RCT	Essential amino acid	44	80.8 ± 7.1	78.9 ± 6.3	Recovery phase	Rehabilitation therapies (physical, occupational, speech)	8
Ikeda T et al., 2020 (32)	RCT	Essential amino acid	46	65.5 ± 13.1	67.5 ± 5.0	Not described	Each training session includes PT and OT for 20 min each, 2 training sessions per day for 2 months	8
Aquilani R et al., 2009 (35)	RCT	Antioxidant	26	72.0 ± 6.5	74.0 ± 8.0	Subacute phase	Routine rehabilitation protocols (Not specifically described)	4
Garbagnati F et al., 2009 (37)	RCT	Antioxidant	72	61.4 ± 13.6	68.5 ± 12.6	Subacute and recovery phase	Individual physiotherapy began within 24 h of admission, lasting 60 min twice daily (once on Saturdays), 6 days a week. After discharge, patients continued with home or outpatient rehab, attending three 1 h sessions weekly.	48

*All patients in all groups received intervention on the basis of routine spontaneous alimentation.

## 3 Results

Patients with ischemic stroke exhibit a high prevalence of malnutrition or are at significant risk for developing malnutrition. It is imperative to conduct nutritional risk screening and initiate early intervention promptly upon hospital admission. Emphasis should be placed on the nutritional management and support of these patients (16). Numerous randomized controlled trials (RCTs) have shown that providing nutritional support to stroke patients can significantly improve rehabilitation outcomes, such as improving limb mobility and cognitive levels in post-stroke patients, reducing infection risks, and NIHSS scores.

### 3.1 Protein

Whether protein supplementation can enhance neurological recovery in patients with subacute ischemic stroke, a randomized controlled trial (RCT) was conducted. The study randomly assigned diet-independent ischemic stroke patients to either a 21-day protein supplementation group (*n* = 20) or a natural diet control group (*n* = 21). Neurological recovery was assessed using the National Institutes of Health Stroke Scale (NIHSS). The findings indicated that increased protein intake was associated with a reduction in NIHSS scores among post-stroke patients (21). Another RCT showed that malnourished stroke patients receiving enhanced nutritional supplementation had improved motor function (total Functional Independence Measure (FIM) scores, FIM motor subscores, 2 min and 6 min timed walk tests, all significant *p* < 0.002) compared with patients receiving standard supplementation; however, there was no improvement in cognitive measures (FIM cognitive score) (22). Similar findings were reported in another RCT, which indicated that aerobic exercise training combined with protein supplementation can significantly enhance balance and motor coordination in stroke patients, as well as improve their daily living abilities (23).

A meta-analysis (RCT = 8, *n* = 5,484) published in 2019 demonstrated that, in comparison to rehabilitation alone, the integration of high-energy, high-protein dietary supplementation with rehabilitation significantly reduces the risk of infections, including pneumonia. However, this intervention did not yield a significant improvement in activities of daily living (ADL) (24). Further research, comprising more extensive and higher-quality randomized controlled trials, is necessary to elucidate the effects of protein supplementation on the rehabilitation outcomes of post-stroke patients (Table 2).

**Table 2:** Impact of protein supplements on stroke patient rehabilitation.

Authors, Year	Intervention measure		Duration of intervention (week)	Outcome measures	Conclusions
	Experimental	Control			
Aquilani R et al., 2008 (21)	Daily intake of protein-rich supplement (250 Kcal energy, 20 g proteins, 28 g carbohydrates and 7 g lipids)	Blank control	3	NIHSS score^▲^	Protein supplementation enhances neurological recovery in patients with subacute ischemic stroke
Rabadi MH et al., 2008 (22)	Take the supplement every 8 h (240 calories, 11 g protein).	Take the placebo every 8 h (127 calories, 5 g of protein).	2	FIM total score^▲^; FIM exercise score^▲^; 2MWT or 6MWT^▲^; Discharge rate to home^▲^; FIM cognitive score	Enhanced nutritional supplementation led to better motor function improvements in patients.
Cheng YH et al., 2020 (23)	Each patient consumed 20 g of a protein supplement (23.2 g protein, 11 g carbohydrates, 144.2 kcal) before and after each training session.	Each patient took 20 g of control supplement (35.2 g carbs, 0.2 g protein) before and after each training session.	8	TUGT; 6MWT; Berg Balance Scale Score^▲^; LE-FMA; Barthel Index	Aerobic exercise training combined with protein supplementation significantly improves balance in stroke patients
Sakai K et al., 2019 (24)	Energy and/or protein	Placebo or blank control	_	ADL; All-cause mortality; Infection incidence^▲^	Protein and energy supplementation did not demonstrate a statistically significant impact on activities of daily living (ADL); however, it was associated with a reduction in the incidence of infection.

^▲^: Indicates a statistically significant difference between the intervention and control groups (*p* < 0.05). FIM, functional independence assessment; 6MWT, 6-minute walking test; 2MWT, 2-minute walking test; ADL, activity of daily living.

### 3.2 Vitamins D

Researchers suggest vitamin D supplements aid stroke recovery, with low levels linked to worse outcomes. An RCT study split ischemic stroke patients with low vitamin D into two groups: one received 300,000 IU vitamin D injections, the other got saline injections. Over 3 months, their recovery was assessed using the Brunnstrom Recovery Stage, Functional Walking Scale, Modified Barthel Index, and Berg Balance Scale. The results indicated that vitamin D supplementation significantly improved patients’ Berg Balance Scale and Modified Barthel Index scores compared to the control group, but had no significant impact on the Brunnstrom Recovery Stage or Functional Walking Scale. This suggests that vitamin D enhances balance and activity levels but does not affect walking or motor recovery (25). Another RCT study showed that Vitamin D supplementation can significantly improve SSS scores, reduce neurological deficits, and promote recovery in patients after a stroke. Furthermore, another RCT indicated that vitamin D supplementation can significantly enhance Scandinavian Stroke Scale (SSS) scores, reduce neurological deficits, and facilitate recovery in post-stroke patients (26).

However, the findings of another multicenter, randomized, double-blind, placebo-controlled trial presented contrasting results. In this study, 100 patients admitted to a rehabilitation ward following an acute stroke were randomly assigned to receive either vitamin D3 (2000 IU/day) or a placebo for 8 weeks. The outcomes indicated that oral vitamin D3 supplementation did not enhance the Barthel index score, Barthel index efficiency, hand grip strength, or calf circumference post-stroke, and had no significant impact on rehabilitation outcomes (27). Additionally, oral vitamin D supplementation did not significantly improve limb function recovery or alleviate depression in post-stroke patients. This was further examined in a 12-week randomized, double-blind, parallel, single-center clinical trial. Participants in the experimental group received a daily oral dose of 2000 IU of cholecalciferol, while the control group did not receive any vitamin D supplementation. Psychological and motor outcomes were assessed through standardized text evaluations. The findings indicated that vitamin D supplementation did not exert a significant impact on mood or functional recovery during the stroke rehabilitation period (28).

Although vitamin D supplementation has not been shown to improve muscle strength and activities of daily living, oral vitamin D supplementation has been shown to inhibit fat infiltration into muscle. A randomized controlled trial (RCT) involving 50 participants examined the impact of nutritional supplementation with whey protein and vitamin D on muscle mass and muscle quality in post-stroke patients. This single-blind, placebo-controlled study allocated the 50 patients into two groups: the high-protein (HP) group received a supplemented jelly containing 100 kcal, 10 g of whey protein, and 20 μg of vitamin D, administered twice daily for up to 16 weeks, while the control group received a placebo jelly. The cross-sectional area (CSA) of the thigh muscle, skeletal muscle index (SMI), muscle strength, activities of daily living (ADL), and some nutritional indicators in the blood were measured. Although there were no significant differences in CSA and SMI between the two groups, fat infiltration of the thigh muscle was significantly lower in the HP group. There were no significant differences in muscle strength and ADL between the two groups. Whey protein and vitamin D supplementation in post-stroke patients can inhibit fat infiltration into muscle (29) (Table 3).

**Table 3:** Impact of vitamins D supplements on stroke patient rehabilitation.

Authors, Year	Intervention measure		Duration of intervention (week)	Outcome measures	Conclusions
	Experimental	Control			
Aylin Sari, 2018 (25)	Upon admission, 2 mL of fluid containing 300,000 IU of vitamin D was injected intramuscularly once	Upon admission, 2 mL saline was injected intramuscularly once	12	BRS; FAS score; MBI score^▲^; BBS score^▲^	Vitamin D supplementation boosted activity and sped up balance recovery but did not significantly impact walking or locomotor recovery.
Shuba Narasimhan, 2017 (26)	60,000 IU of cholecalciferol intramuscularly at time of admission	Blank control	12	Scandinavian Stroke Scale (SSS)^▲^	Vitamin D supplementation can significantly improve SSS scores, reduce neurological deficits, and promote recovery in patients after a stroke.
Momosaki R et al., 2019 (27)	Vitamin D supplementation (2000 IU/day vitamin D3)	Placebo	8	Barthel Index scores; Barthel Index efficiency (Brunnstrom stages; grip strength; calf circumference)	Oral supplementation with vitamin D3 does not enhance rehabilitation outcomes following an acute stroke.
Torrisi M et al., 2021 (28)	vitamin D supplementation (2000IU/day of oral cholecalciferol)	Blank control	12	GSE score; MADRS score; FIM	Vitamin D supplementation does not enhance functional recovery or mood in individuals recovering from a stroke.
Honaga K et al., 2022 (29)	Take supplemental jelly twice daily (100 kcal, 10 g whey protein, 20 μg vitamin D).	Blank control	16	SMI; CSA of the thigh muscles; muscle strength; ADL; Fatty infiltration of thigh muscles^▲^	Whey protein and vitamin D supplements prevent muscle fat infiltration in post-stroke patients.

^▲^: Indicates a statistically significant difference between the intervention and control groups (*p* < 0.05). GSE, General Self Efficacy Scale; MADRS, Montgomery Asberg Depression Rating Scale; FIM, functional independence assessment; SMI, skeletal muscle index; CSA, cross-sectional area; ADL, activity of daily living; BRS, Brunnstrom recovery staging; FAS, functional ambulation scale; MBI, modified Barthel index; BBS, Berg balance scale.

### 3.3 Essential amino acids

Elevated concentrations of essential amino acids, including glutamate, aspartate, and *γ*-aminobutyric acid, alongside reduced levels of glycine in plasma, are correlated with unfavorable prognoses following ischemic stroke. This observation indicates that plasma amino acid neurotransmitters may serve as viable targets for intervention aimed at enhancing outcomes in ischemic stroke patients (30). A randomized controlled trial (RCT) was conducted involving an eight-week, two-group parallel intervention with randomized control and blinded outcome assessment in 44 elderly post-stroke patients diagnosed with sarcopenia. The findings indicated that supplementation with leucine-rich amino acids led to significant improvements in Functional Independence Measure (FIM) scores and grip strength among the post-stroke patients. However, there was no significant improvement observed in the skeletal muscle mass index (SMI) (31).

The timing of essential amino acid supplementation may also facilitate recovery. A randomized controlled trial (RCT) examined the effects of the timing of branched-chain amino acid (BCAA) supplementation combined with exercise intervention on physical function in stroke patients. Participants were randomized into two groups based on the timing of supplementation: breakfast (*n* = 23) and post-exercise (*n* = 23). In the breakfast group, supplementation was administered at 08:00, whereas in the post-exercise group, supplementation was provided immediately after exercise between 14:00 and 18:00. In both cohorts, the exercise intervention was administered bi-daily over a two-month period. The findings indicated that the timing of supplementation had a comparable impact on skeletal muscle mass across both groups. However, the ingestion of branched-chain amino acids (BCAAs) at breakfast was particularly efficacious in enhancing physical function and decreasing body fat mass. These results imply that the integration of BCAA consumption at breakfast with a structured exercise regimen is effective in facilitating recovery in post-stroke patients (32) (Table 4).

**Table 4:** Impact of essential amino acids supplements on stroke patient rehabilitation.

Authors, Year	Intervention measure		Duration of intervention (week)	Outcome measures	Conclusions
	Experimental	Control			
Yoshimura Y et al., 2019 (31)	Take the leucine-rich amino acid supplement (This supplement contains 3 grams of essential amino acids rich in 40% leucine) once daily.	Blank control	8	ADL^▲^; SMI^▲;^ HGS^▲^	Leucine-rich supplements and low-intensity resistance training enhance muscle mass, strength, and physical function in post-stroke sarcopenia patients.
Ikeda T et al., 2020 (32)	A leucine-enriched nutritional supplement (3.5 g of amino acids and 6.5 g of protein, along with 40 IU of vitamin D per 125 mL) provided with breakfast	The same supplement is provided immediately after the workout from 14:00–18:00 in the afternoon	8	Skeletal muscle mass; Leg press strength^▲^; Grip strength; Body fat mass^▲^; BBS^▲^; TUGT; FIM	Consuming a leucine-enriched nutritional supplement during breakfast demonstrates greater efficacy in enhancing physical function and reducing body fat compared to its consumption following rehabilitation exercise.

^▲^: Indicates a statistically significant difference between the intervention and control groups (*p* < 0.05). ADL, activity of daily living; SMI, skeletal muscle index; HGS, handgrip strength; BBS, Berg balance scale; TUGT, timed up-and-go test; FIM, functional independence assessment.

### 3.4 Antioxidants

Oxidative stress and inflammation contribute significantly to the cascade of stroke-related malnutrition and ischemic events in the brain. Ischemic damage results in neuronal death and cerebral infarction, which, through intercellular signaling, disrupt the neuroplasticity processes essential for functional recovery facilitated by multidisciplinary rehabilitation therapy. Nutritional interventions incorporating food components with antioxidative and anti-inflammatory properties have the potential to mitigate or prevent post-stroke malnutrition. These strategies may be essential for enhancing neuroplasticity, thereby facilitating improved rehabilitation outcomes in stroke patients (33).

Zinc is an essential trace element for human survival. Zinc plays a key role in neuronal proliferation, differentiation, neuronal migration and axonal growth (34). Improving neurological function after stroke through zinc supplementation is a promising therapeutic strategy. A randomized controlled trial (RCT) was conducted to investigate the potential contribution of zinc supplementation to neurological recovery in patients with stroke and low zinc intake. Twenty-six patients with subacute stroke were randomly assigned to either a control group or a zinc supplementation group, with the latter receiving 10 mg/day of zinc. After 30 days of treatment, neurological severity was assessed using the NIH Stroke Scale (NIHSS). The findings indicated that the improvement in NIHSS scores was significantly greater in the zinc supplementation group compared to the placebo group. These results suggest that normalizing zinc intake may enhance neurological recovery in stroke patients with initially low mineral intake (35). The results of animal experiments indicate that injecting the zinc chelator ZnEDTA 14 days after middle cerebral artery occlusion (MCAO) in adult male rats significantly reduced infarct volume and neuronal damage and improved neurological function (36).

A randomized, double-blind, placebo-controlled clinical trial demonstrated that n-3 polyunsaturated fatty acids can reduce the 1-year mortality rate in post-stroke patients. However, these fatty acids did not show significant improvement in neurological function, as measured by the Canadian Neurological Scale (CNS), nor in daily living activities, as assessed by the Barthel Index (BI) and Rivermead Mobility Index (RMI). The study involved the random allocation of 72 patients (47 males; mean age 65.3 ± 12.9 years), who were admitted to a rehabilitation hospital for the sequelae of their first ischemic stroke, into four subgroups. Patients in Group 1 received daily oral antioxidants (including: 290 mg vitamin E, 240 mg vitamin C, 150 mg polyphenols and 19 mg carotene), Group 2 received n-3 polyunsaturated fatty acids, Group 3 received both supplements, and Group 4 received a placebo, all for a duration of 12 months. The results indicated a trend towards a lower mortality rate in the subgroup treated with n-3 fatty acids at the 1-year follow-up (*p* = 0.060). However, there were no significant differences in rehabilitation outcomes, as measured by neurological function (Canadian Neurological Scale, CNS) and activities of daily living (Barthel Index, BI; Rivermead Mobility Index, RMI), between the groups (37) (Table 5).

**Table 5:** Impact of antioxidants supplements on stroke patient rehabilitation.

Authors, Year	Intervention measure		Duration of intervention (week)	Outcome measures	Conclusions
	Experimental	Control			
Aquilani R et al., 2009 (35)	10 mg of elemental Zn2+ was given at 10 am daily.	Placebo	4	NIHSS score^▲^	Zinc supplementation (Zn2+) lowers NIHSS scores and aids neurological recovery in stroke patients.
Garbagnati F et al., 2009 (37)	Antioxidant (290 mg vitamin E, 240 mg vitamin C, 150 mg polyphenols and 19 mg carotene) once daily	Placebo	48	1-year mortality rate^▲^; Canadian Neurological Scale; Barthel Index; Rivermead Mobility Index	Oral antioxidants do not enhance limb function in post-stroke patients but significantly lower 1-year mortality.

^▲^: Indicates a statistically significant difference between the intervention and control groups (*p* < 0.05).

## 4 Discussion

Nutritional intervention plays a crucial role in the rehabilitation process for stroke patients. Post-stroke individuals frequently experience malnutrition, which adversely affects their physical health and impedes their rehabilitation progress (38). Consequently, nutritional intervention is essential during the rehabilitation phase. This article elucidates the substantial influence of nutrient supplementation, including protein, essential amino acids, vitamin D, and antioxidants, on the rehabilitation outcomes of stroke patients.

Currently, pharmacological interventions for cerebral infarction primarily focus on enhancing cerebral microcirculation. However, a significant proportion of patients continue to experience varying degrees of neurological dysfunction, including hemiplegia and aphasia (39). While acute thrombolysis has demonstrated efficacy, its application is constrained by a narrow therapeutic window of 3 to 6 h, thereby limiting its availability to a small subset of patients (40, 41). Consequently, the majority of individuals with cerebral infarction are likely to endure persistent neurological impairments.

Hemiplegic stroke can result in a range of muscle abnormalities, characterized by a complex interplay of denervation, disuse, inflammation, remodeling, and spasticity, which collectively lead to phenotypic changes in muscle tissue and atrophy (42). The onset of muscle atrophy following a stroke significantly impedes the patient’s rehabilitation process. Research indicates that the muscle thickness and architecture of the lower leg muscles, such as the pennation angle (PA) and fascicle length (FL), are markedly reduced on the affected side compared to the unaffected side in stroke patients. Specifically, the thickness of the soleus and gastrocnemius muscles, as well as the pennation angle of the gastrocnemius, are associated with balance and motor function (43). These findings are corroborated by a meta-analysis (RCT = 15, *n* = 375), which identified stroke-related sarcopenia as a contributing factor to muscle dysfunction on the hemiplegic side (44). High protein intake, particularly through whey protein or branched-chain amino acid supplements, has been shown to significantly enhance skeletal muscle protein synthesis and improve muscle tissue quality. Research indicates that amino acids can mitigate excessive muscle breakdown in post-stroke patients by inhibiting the degradation of myofibrillar protein and skeletal muscle. Supplementation with amino acids can prevent muscle atrophy and facilitate rehabilitation by enhancing physical function, muscle strength, and muscle quality and function (45). It is well-established that therapeutic exercise is frequently employed in exercise rehabilitation programs for stroke patients to enhance physical function. Through structured limb exercises and appropriate activities, patients can improve muscle strength, cardiopulmonary endurance, walking ability, and daily living skills, thereby achieving the goals of injury prevention, functional improvement, and overall health promotion (46). Research indicates that rehabilitation exercise training plays a crucial role in maintaining brain function, enhancing brain plasticity, and increasing resistance to cerebral damage such as ischemia. Prompt and proactive exercise rehabilitation following a stroke has been shown to reduce cerebral infarction volume and ischemia-induced neuronal apoptosis, facilitate the remodeling of corticospinal neurons, and promote axonal growth and dendritic branching, thereby aiding neurological function recovery (47–49). Additionally, some studies have explored the integration of rehabilitation exercise with protein or amino acid supplementation, revealing that such supplementation enhances the adaptive effects and efficacy of exercise rehabilitation (50, 51), which aligns with our findings. Rehabilitation exercise contributes to muscle tissue growth and improved muscle strength. During the rehabilitation phase, protein or amino acid supplementation provides essential substrates for muscle tissue growth, significantly enhancing the efficiency of muscle protein synthesis and improving muscle quality, thus playing a synergistic role in promoting patient recovery.

Patients experiencing residual limb dysfunction post-stroke may exhibit diminished vitamin D synthesis due to prolonged bed rest and limited exposure to sunlight and ultraviolet radiation, rendering them more vulnerable to vitamin D deficiency. Beyond its role in regulating calcium and phosphorus metabolism, vitamin D also exerts numerous non-calcemic effects, including immunomodulation, neuroprotection, oxidative stress inhibition, and the regulation of cellular proliferation and apoptosis (52). The biologically active form of vitamin D, 1,25-dihydroxyvitamin D (1,25(OH)₂D), primarily functions through its interaction with the vitamin D receptor (VDR). Research indicates that following cerebral ischemia, there is a significant upregulation of VDR expression in microglia/macrophages surrounding the infarct area. Inactivation of VDR in these cells has been shown to markedly increase infarct volume and exacerbate neurological deficits. Microglia/macrophages deficient in VDR exhibit a pronounced pro-inflammatory phenotype, characterized by the secretion of elevated levels of TNF-*α* and IFN-*γ*. Supplementation with 1,25(OH)₂D, a bioactive form of vitamin D known to activate the vitamin D receptor (VDR), has been shown to effectively enhance VDR expression and suppress the expression of ischemia-induced cytokines such as TNF-α and IFN-γ, thereby mitigating secondary brain damage (53). Additionally, another study demonstrated an association between lower serum levels of 1,25(OH)₂D and an increased risk of recurrent stroke in patients with ischemic stroke (54). The present study revealed that, compared to rehabilitation therapy alone, the administration of high-dose vitamin D via intramuscular injection, in conjunction with rehabilitation therapy upon admission, significantly ameliorated neurological deficit symptoms in patients. Conversely, oral administration of low-dose vitamin D combined with rehabilitation therapy did not yield significant improvements in the neurological deficits of stroke patients. Consequently, further research is warranted to ascertain whether varying doses and methods of vitamin D administration differentially impact the amelioration of neurological deficits in stroke patients.

Following a stroke, vascular occlusion resulting from ischemia leads to the excessive production of reactive oxygen species (ROS), with oxidative stress being implicated in exacerbating neuronal damage and contributing to significant functional impairments (55). Oxidative stress is regarded as a critical environmental factor that adversely impacts neurogenesis by inhibiting all stages of adult neurogenesis (56, 57). This study demonstrates that the combination of antioxidants and rehabilitation therapy can decrease the NIHSS score, ameliorate neurological deficit symptoms, and reduce 1-year mortality rates in post-stroke patients. The potential mechanism underlying these improvements may involve rehabilitation-induced neural structural changes, such as neural sprouting, synapse formation, and dendritic branching (58). Antioxidants, including polyunsaturated fatty acids (such as *ω*-3 and DHA) and Zn2+, have been shown to mitigate oxidative stress and neuroinflammation, enhance cellular signaling, activate autophagy, and influence growth factors. These compounds facilitate cellular repair and survival by inducing and activating nutritional factors, antioxidant enzymes, DNA repair enzymes, and proteins associated with mitochondrial biogenesis. This process enhances the brain’s resilience to more intense stress when exposed to heightened stimulation (59–61). Consequently, the integration of antioxidants with rehabilitative exercise may promote cerebral remodeling and ameliorate neurological deficits following a stroke.

This study investigated the synergistic effects of rehabilitation exercise and nutritional supplementation, including protein, essential amino acids, vitamin D, and antioxidants, on enhancing exercise rehabilitation adaptation and performance. The findings suggest that various nutritional supplementation strategies can facilitate improvements in these areas. Nonetheless, the study has certain limitations. Firstly, the range of nutrients examined is restricted. Future research will aim to assess the impact of a broader spectrum of nutritional supplements on the rehabilitation of stroke patients. Secondly, the study did not address the treatment of dysphagia. Further research is required to develop nutritional supplementation plans and rehabilitation strategies specifically tailored for stroke patients experiencing dysphagia.
