# Supplementary material for: Influence of nutritional status on rehabilitation efficacy of patients after stroke—a scoping review
Source: Front Neurol. 2025 Jan 29;16:1502772. doi: 10.3389/fneur.2025.1502772 (PMC11813746; doi:10.3389/fneur.2025.1502772)
Supplement: Supplementary file 1 [file Data_Sheet_1.PDF]

Table 1: Impact of protein supplements on stroke patient rehabilitation.

Authors，Year	Design	Number of participants	Age（years）		Stroke course(week)	Duration of intervention(week)	Outcomes
			Intervention	Control			
Aquilani R et al.，2008[26]	RCT	41	71±6.9	68±9.1	4W	3W	NIHSS score▲
Rabadi MH et al., 2008[27]	RCT	116	75.0±10.5	73.58±13.02	4W	2-4W	FIM total score▲; FIM exercise score▲; 2MWT or 6MWT▲; Discharge rate to home▲; FIM cognitive score
Sakai K et al., 2019[28]	Meta-analysis of 8 RCTs	5484	-	-	72h-12W	Not reported	ADL; All-cause mortality; Infection incidence▲

▲: Indicates a statistically significant difference between the intervention and control groups ($p < 0.05$).

FIM=Functional Independence Assessment; 6MWT=6-Minute Walking Test; 2MWT=2-Minute Walking Test; ADL=Activity of Daily Living.

Table 2: Impact of Vitamins D supplements on stroke patient rehabilitation.

Authors，Year	Design	Number of participants	Age（years）		Stroke course(week)	Duration of intervention(week)	Outcomes
			Intervention	Control			
Momosaki R et al.，2019[30]	RCT	97	67.6±11.7	65.5±11.7	4W	8W	Barthel Index scores; Barthel Index efficiency（Brunnstrom stages，grip strength, calf circumference）
Torrisi M et al.，2021[31]	RCT	29	59.20±11.38	62.07±10.82	4-8W	12W	GSE score; MADRS score; FIM
Honaga K et al.，2022[32]	RCT	45	64.2±8.9	61.3±11.5	2--24W	16W	SMI; CSA of the thigh muscles; muscle strength; ADL; Fatty infiltration of thigh muscles▲
Aylin Sari,，2018[29]	RCT	47	69.84±10.09	66.930±10.05	8-24W	12W	BRS; FAS score; MBI score▲; BBS score▲

▲: Indicates a statistically significant difference between the intervention and control groups ($p < 0.05$).

GSE=General Self Efficacy Scale; MADRS=Montgomery Asberg Depression Rating Scale ; FIM=Functional Independence Assessment; SMI= skeletal muscle index; CSA=cross-sectional area; ADL=Activity of Daily Living; BRS=Brunnstrom recovery staging; FAS=functional ambulation scale; MBI=modified Barthel index; BBS=Berg balance scale.

Table 3: Impact of Essential amino acids supplements on stroke patient rehabilitation.

Authors，Year	Design	Number of participants	Age（years）		Stroke course(week)	Duration of intervention(week)	Outcomes
			Intervention	Control			
Yoshimura Y et al.，2019[34]	RCT	44	80.8 ±7.1	78.9±6.3	＞24W	8W	ADL▲; SMI▲; HGS▲
Ikeda T et al.，2020[35]	RCT	46	65.5±13.1	67.5±5	Not reported	8W	skeletal muscle mass、Leg press strength▲; grip strength; Body fat mass▲; BBS▲; TUGT; FIM

▲: Indicates a statistically significant difference between the intervention and control groups ($p < 0.05$).

ADL=Activity of Daily Living; SMI= skeletal muscle index; HGS=Handgrip Strength; BBS=Berg balance scale; (TUGT)=timed up-and-go test; FIM=Functional Independence Assessment.
